# Asymptomatic Achilles tendon pathology is associated with a central fat distribution in men and a peripheral fat distribution in women: a cross sectional study of 298 individuals

**DOI:** 10.1186/1471-2474-11-41

**Published:** 2010-03-02

**Authors:** James E Gaida, Håkan Alfredson, Zoltan S Kiss, Shona L Bass, Jill L Cook

**Affiliations:** 1 School of Exercise and Nutrition Sciences, Deakin University, Burwood, Australia; 2 Sports Medicine Unit, Umeå University, Umeå, Sweden; 3 Victoria House Medical Imaging, Prahran, Australia

## Abstract

**Background:**

Adiposity is a modifiable factor that has been implicated in tendinopathy. As tendon pain reduces physical activity levels and can lead to weight gain, associations between tendon pathology and adiposity must be studied in individuals without tendon pain. Therefore, the purpose of this study was to determine whether fat distribution was associated with asymptomatic Achilles tendon pathology.

**Methods:**

The Achilles tendons of 298 individuals were categorised as normal or pathological using diagnostic ultrasound. Fat distribution was determined using anthropometry (waist circumference, waist hip ratio [WHR]) and dual-energy x-ray absorptiometry.

**Results:**

Asymptomatic Achilles tendon pathology was more evident in men (13%) than women (5%) (p = 0.007). Men with tendon pathology were older (50.9 ± 10.4, 36.3 ± 11.3, p < 0.001), had greater WHR (0.926 ± 0.091, 0.875 ± 0.065, p = 0.039), higher android/gynoid fat mass ratio (0.616 ± 0.186, 0.519 ± 0.142, p = 0.014) and higher upper-body/lower body fat mass ratio (2.346 ± 0.630, 2.022 ± 0.467, p = 0.013). Men older than 40 years with a waist circumference >83 cm had the greatest prevalence of tendon pathology (33%). Women with tendon pathology were older (47.4 ± 10.0, 36.0 ± 10.3, p = 0.008), had less total fat (17196 ± 3173 g, 21626 ± 7882 g, p = 0.009), trunk fat (7367 ± 1662 g, 10087 ± 4152 g, p = 0.003) and android fat (1117 ± 324 g, 1616 ± 811 g, p = 0.005). They had lower central/peripheral fat mass ratios (0.711 ± 0.321 g, 0.922 ± 0.194 g, p = 0.004) than women with normal tendons. Women with tendon pathology were more often menopausal (63%, 13%, p = 0.002).

**Conclusions:**

Men with Achilles tendon pathology were older and had a central fat distribution. Women with tendon pathology were older and had a peripheral fat distribution. An interaction between age and waist circumference was observed among men.

## Background

Achilles tendinopathy is a common injury and causes pain during activities that place load on the Achilles tendon -- activities such as standing, walking, jogging and running [1]. Not surprisingly, the condition is most common among those with high levels of tendon loading -- 1 in every 2 runners will experience Achilles tendinopathy before the age of 45 [2]. Surprisingly, people with low levels of tendon loading are also frequently affected -- among the general community 1 in every 10 persons will be affected within their lifetime [2]. This condition prevents individuals from participating in physical activity, precluding the benefits of physical activity on health and obesity.

Treatment for Achilles tendinopathy can be lengthy and frustrating [3], and, in the process, costly to the individual and to the health system. As such, effective prevention should be a priority. Identification of risk factors is a critical first step in developing prevention programmes. Recent evidence suggests that the amount and distribution of adiposity may be a risk factor for tendinopathy [4,5]. As greater adiposity is amenable to change through diet and exercise interventions, this factor could potentially be incorporated into prevention programmes.

A recent systematic review highlighted the association between increased adiposity and tendinopathy [5]. This review included all published papers addressing tendinopathy that also included a valid measurement of adiposity. In 43% of cases, the group with tendinopathy had significantly greater adiposity levels than the control group without tendinopathy. Two other key findings of that review are worth repeating. First, when upper-limb and lower-limb tendinopathies were compared the association with adiposity was equally strong. As the lower-limb tendons support body-weight while the upper-limb tendons support only the weight of the limb, this finding suggested that mechanical loading does not fully explain the association between adiposity and tendinopathy. Second, the longitudinal studies in the review that showed baseline adiposity predicted tendinopathy at follow-up suggested that adiposity is a risk factor for tendinopathy rather than a consequence of tendinopathy.

Specifically, the data showed that the distribution of adipose tissue is related to patellar tendon pathology in lean male athletes [6] and lean female athletes [7], and to medial elbow tendinopathy in male and female residents of Finland [8]. These studies suggest that a central accumulation of adipose tissue is harmful to tendons. Adding to this argument is the recent finding that individuals with painful mid-portion Achilles tendinopathy display a dyslipidaemia characteristic of insulin resistance [9]. As both dyslipidaemia and insulin resistance are closely related to the expansion of intra-abdominal (i.e. visceral) adipose tissue depots [10,11], precise measurement of adipose tissue storage and exploration of its association with Achilles tendon pathology is warranted.

Previous studies that have investigated adiposity and symptomatic tendinopathy have not been able to avoid the confounding effect of pain. Achilles tendon pain leads to decreases in physical activity, and such reductions in physical activity are known to lead to increases in adiposity [12,13]. Indeed, our findings show that 40% of patients with Achilles tendinopathy report gaining weight since their tendon first became painful, and among this group their average estimated weight gain was 5 kg (Gaida et al. unpublished data). To avoid this confounding factor, all participants should be free from pain when studying associations between adiposity and Achilles tendon pathology. Diagnostic ultrasound can be used to detect tendon pathology in individuals that have no tendon pain [14]. Thus, the aim of this investigation was to compare regional adipose tissue distribution between members of the general community with asymptomatic Achilles tendon pathology and those with normal Achilles tendons. Our hypothesis was that a central accumulation of adipose tissue would be related to Achilles tendon pathology, and that the association would be strongest in men.

## Methods

Participants in this study were healthy and had no current or previous symptoms of Achilles tendinopathy. Pregnant or breast-feeding women were not eligible as the study involved exposure to ionising radiation. Women were asked to self report whether they were pre-menopausal, peri-menopausal (≥ 1 menstrual cycle in last 12 months) or post-menopausal (greater than 12 months since last menstrual cycle). Members of the general population were invited to participate in this study via advertisements posted in public locations and through email contact. Participants were asked to forward the details of the study to other potential participants. A university ethics committee approved the study and all participants gave informed consent.

Data were collected with comparable methods from two locations -- Australia (97 men, 146 women) and Sweden (30 men, 25 women). Participants aged 18 to 75 years were eligible. To avoid bias, researchers measuring anthropometric and fat-distribution variables were unaware of the results of the ultrasound examination.

### Anthropometry

The same registered anthropometrist (JEG) measured anthropometric variables in all participants (Australian and Swedish) according to published protocols [15]. All anthropometric variables were measured twice, with the final result taken as the mean of the two measures. If the measurement error exceeded 1%, a third measurement was performed and the median value taken as the final result.

A wall-mounted stadiometer (Australian Group -- [Heightronic 235; Measurement Concepts, North Bend, Washington, USA], Swedish Group -- [Harpenden Stadiometer, Holtain Ltd, Crymych, Dyfed, UK]) was used to record standing height to the nearest millimetre. Weight was measured in light clothing and with shoes removed using an electronic scale (Australian Group -- [UC-321; A&D Mercury Pty Ltd, Adelaide, South Australia, Australia], Swedish Group -- [HL 120, Avery Berkel, Smethwick, UK]).

Waist circumference was measured using a flexible steel tape (Lufkin W606PM; Cooper Hand Tools, Raleigh, North Carolina, USA) against bare skin. Waist circumference was measured to the nearest millimetre in a horizontal plane at the midpoint between the iliac crest and lower costal margin (palpated in the mid axillary line). Repeated measurement of waist circumference was performed on 20 subjects. The technical error of measurement (TEM) for JEG was calculated at 0.45% and the intra-class correlation coefficient (ICC) was calculated at ICC(3,1) = 0.998. Hip circumference was measured in a horizontal plane at the level of the greatest posterior protuberance of the gluteal muscles as viewed from the side. Repeated measurement of hip circumference was performed on 20 subjects. The TEM for JEG was calculated at 0.29% and ICC(3,1) = 0.998.

### Dual-Energy X-ray Absorptiometry

A trained and licenced bone densitometrist performed full body scans using a dual-energy x-ray absorptiometer (DXA) (Australian Group -- [Lunar Prodigy (GE Lunar Radiation Corporation, Madison, Wisconsin, USA) using the multimedia version of EnCore software 8.10.027], Swedish Group -- [DPX-IQ (GE Lunar Radiation Corporation, Madison, Wisconsin, USA) using *Smart Scan *software version 4.7e]). Quality control was performed daily before scanning the first subject using a phantom. After acquisition, all scans were analysed in an identical manner by a bone densitometrist who was blind to group allocation. This study used standard DXA regions of interest (ROI); arms, legs, trunk, android, gynoid.

**Figure 1:**
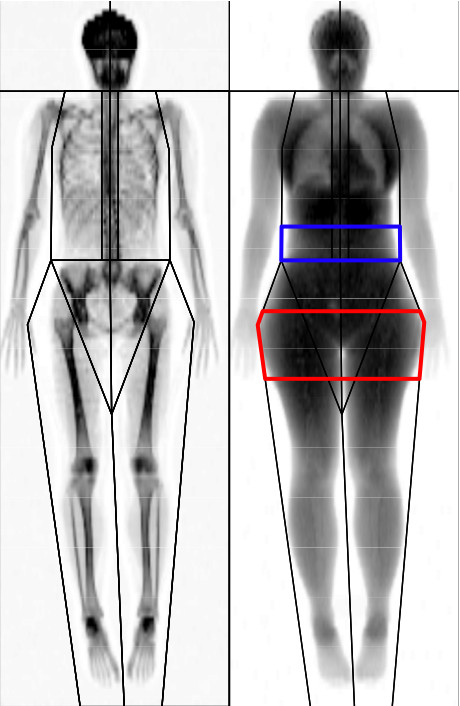
**Typical full body DXA scan**. The android region of interest (ROI) is highlighted in blue and the gynoid ROI is highlighted in red (refer to text for the landmarks that define these regions).

The android ROI (Figure 1 -- shown in blue) is representative of where many men preferentially store excess body fat. The base of the android ROI sits immediately above the pelvis and is equal in height to 20% of the distance from the pelvis to the chin. The gynoid ROI (Figure 1 -- shown in red) is representative of where many women preferentially store excess body fat. The android and gynoid ROI are separated by a distance equal to 1.5 times the height of the android ROI, while the height of the gynoid ROI is double that of the android ROI.

As Australian and Swedish data were collected on different machines, inter-machine reliability is critical. The machine models used here have previously been cross-validated and agree very closely (total body fat r^2 ^= 0.991) [16]. The ROIs were defined using the same landmarks for both machines. The repeatability of measurement on the DPX-IQ (which does not automatically calculate the android and gynoid ROIs) was determined by reanalysing 20 scans (10 men, 10 women); android fat ICC(3,1) = 1.000 (95% CI 0.999 to 1.000), gynoid fat ICC(3,1) = 0.999 (95% CI 0.997 to 1.000).

In addition to standard ROIs, the pattern of adipose tissue distribution was analysed. The android/gynoid fat mass ratio, the upper-body/lower-body fat mass ratio and the central/peripheral fat mass ratio were calculated.

### Ultrasound

An experienced musculoskeletal radiologist (ZSK, Fellow of the Royal Australian and New Zealand College of Radiologists) performed all ultrasound examinations in the Australian group (Acuson Aspen Advanced fitted with 5-10 MHz linear array transducer; Siemens AG, Munich, Germany). An experienced orthopaedic surgeon (HA) performed all ultrasound examinations in the Swedish group (Acuson Sequoia 512 fitted with 8-13 MHz linear array transducer; Siemens AG, Munich, Germany).

The Achilles tendon was examined both longitudinally and transversely to assess the overall structure, such as internal fibre arrangement, blurring or irregularity of tendon borders or bowing of the anterior border of the tendon. During the longitudinal scan, care was taken to ensure that the transducer was parallel to the tendon fibres to avoid false positive findings (anisotropy) [17]. Each tendon was classified as having a normal or abnormal internal structure. A tendon was classified as abnormal if any of the three following conditions were met 1) one or more focal hypoechoic regions visible in both the longitudinal and transverse scans, 2) diffuse hypoechogenicity associated with bowing of the anterior tendon border, or 3) diffuse hypoechogenicity associated with generalised thickening of the tendon in comparison to the contralateral tendon. Highly trained operators have been shown to accurately categorise tendons as normal or abnormal -- (kappa = 1)[18,19]. Additionally, ultrasound is superior to MRI in classifying patellar tendon structure when using an expert clinical diagnosis of patellar tendinopathy as the gold standard [20].

The patellar and supraspinatus tendons were also examined in the Australian participants using the same methods. This data was used to examine whether having an abnormal Achilles tendon was associated with abnormality affecting other tendons.

### Statistics

An *a priori *decision was made to analyse men and women separately, as body composition is sexually dimorphic [21]. Variables measured on a continuous scale were tested for normality (Kolmogorov-Smirnov test). Normally distributed variables were compared between the groups (asymptomatic pathology versus normal) using an unpaired t-test. If the assumption of normality was violated, the Mann-Whitney U test was used. Categorical variables were tested for group differences using the chi-square test.

A secondary analysis of the results was conducted whereby a potential interaction between age and central adiposity was explored. This consisted of a simple scatterplot of age versus waist circumference that was divided into four groups. The data was first divided into two groups according to whether an individual was ≤ 40 or > 40 years of age. This age has previously been used to dichotomise data in tendinopathy research [22] and is similar to the average age of men presenting with symptomatic Achilles tendinopathy [23]. The data was next divided using a waist circumference cut-off. In the men this was guided by previous tendinopathy research [6], which suggested a cut-off of 83 cm. In the lack of guiding data from the tendinopathy literature for the women, the cut-off of 80 cm was selected from the cardiovascular literature. A waist circumference of 80 cm is the designated action level 1 where no further weight should be gain in order to minimised the risk of future cardiovascular events [24]. Clustering of individuals with Achilles tendon pathology into any of these four groups was analysed using a chi-square analysis.

## Results

Data was collected from 298 subjects - 127 men and 171 women (Table 1). Asymptomatic Achilles tendon pathology was evident in more men (17/127, 13%) than women (8/171, 5%) (χ^2 ^= 7.189, df = 1, p = 0.007).

**Table 1:** Demographics (mean (SD)) of study participants.

	Men (n = 127)	Women (n = 171)
Age (years)	38.3 (12.2)	36.5 (10.5)
Height (cm)	179.5 (6.8)	166.6 (6.7)
Weight (kg)	82.3 (11.0)	65.9 (9.3)
BMI (kg/m^2^)	25.6 (3.5)	23.7 (3.2)

### Men

Seventeen men had asymptomatic Achilles tendon pathology; the remaining 110 men had normal Achilles tendon structure. The men with pathology were older than subjects with normal tendons (50.9 ± 10.4 years, 36.3 ± 11.3 years, p < 0.001). Those with pathology had greater WHR (0.926 ± 0.091, 0.875 ± 0.065, p = 0.039), higher android/gynoid fat mass ratio (0.616 ± 0.186, 0.519 ± 0.142, p = 0.014) and higher upper-body/lower body fat mass ratio (2.346 ± 0.630, 2.022 ± 0.467, p = 0.013) (Table 2).

**Table 2:** Anthropometric and body composition (mean (SD)) comparison between men with and without tendon pathology.

		Normal Achilles n = 110	Abnormal Achilles n = 17	p value
Age (years)		36.3 (11.3)	50.9 (10.4)	<0.001
Anthropometry				
	Height (cm)	179.7 (6.9)	177.8 (6.3)	0.282
	Weight (kg)	82.2 (10.9)	83.5 (11.8)	0.657
	BMI (kg/m^2^)	25.5 (3.5)	26.4 (3.2)	0.334
	Waist (cm)	88.4 (9.8)	92.5 (11.6)	0.116
	Hip (cm)	100.9 (6.1)	99.7 (4.5)	0.460
	WHR‡	0.875 (0.065)	0.926 (0.091)	0.039
				
Fat				
	Total (g)	18513 (8317)	20104 (7669)	0.460
	Arms (g)	1543 (854)	1833 (856)	0.195
	Leg (g)	5862 (2487)	5724 (1764)	0.826
	Trunk (g)	10459 (5068)	11729 (5143)	0.339
	Android (g)	1838 (987)	2084 (976)	0.341
	Gynoid (g)	3385 (1340)	3303 (988)	0.808
				
Fat Mass Ratio				
	Android/Gynoid	0.519 (0.142)	0.616 (0.186)	0.014
	Upper-body/Lower-body	2.022 (0.467)	2.346 (0.630)	0.013
	Central/Peripheral‡	1.393 (0.270)	1.533 (0.396)	0.176
Changes in other tendons (y:n)§		11:76	2:8	0.619

† - Variable not normally distributed, Mann-Whitney U test.

‡ - Normally distributed but significant difference in variance between groups.

§ - Expected cell count < 5, Fisher's Exact test.

Men with pathology in the Achilles tendon were no more likely to have pathology of the patellar or rotator cuff tendons (2/10 [20%], 11/87 [13%], p = 0.619) (Table 2).

**Figure 2:**
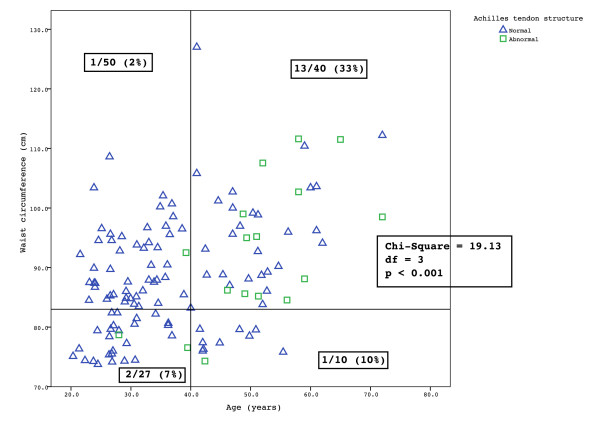
**Scatter-plot of age and waist circumference in men**. Men with normal tendons are indicated by blue triangles while men with asymptomatic Achilles tendon pathology are indicated by green squares.

The scatterplot of age versus waist circumference showed a clustering of men with abnormal tendons in the group aged over 40 years and with a waist circumference above 83 cm (χ^2 ^= 19.13, df = 3, p < 0.001) (Figure 2). In this group the prevalence of tendon pathology was 33% (13/40) whereas in the other groups the prevalence was between 2% and 10%.

### Women

Eight women had asymptomatic Achilles tendon pathology; the remaining 163 female subjects had normal Achilles tendon structure. Women with Achilles tendon pathology were older (47.4 ± 10.0, 36.0 ± 10.3, p = 0.008), shorter (161.7 ± 6.1, 166.8 ± 6.6, p = 0.046), lighter (59.1 ± 7.9, 66.2 ± 9.3, p = 0.048), and were more likely to be peri/post-menopausal (5/8 [63%] versus 21/163 [13%], p = 0.002) than subjects with normal tendons. There was no difference in BMI between the groups (p = 0.319) (Table 3).

**Table 3:** Anthropometric and body composition (mean (SD)) comparison between women with and without tendon pathology.

		Normal Achilles n = 163	Abnormal Achilles n = 8	p value
Age (years)†		36.0 (10.3)	47.4 (10.0)	0.008
Menopause (pre:peri/post)		142:21	3:5	0.002
Anthropometry				
	Height (cm)	166.8 (6.6)	161.7 (6.1)	0.046
	Weight (kg)	66.2 (9.3)	59.1 (7.9)	0.048
	BMI (kg/m^2^)	23.8 (3.2)	22.6 (2.6)	0.319
	Waist (cm)	77.0 (8.8)	75.2 (5.3)	0.596
	Hip (cm)	100.4 (6.8)	95.6 (6.9)	0.065
	WHR‡	0.766 (0.065)	0.789 (0.064)	0.367
				
Fat				
	Total (g)‡	21626 (7882)	17196 (3173)	0.009
	Arms (g)	1957 (882)	1673 (423)	0.399
	Leg (g)	8897 (3058)	7436 (1484)	0.211
	Trunk (g)‡	10087 (4152)	7367 (1662)	0.003
	Android (g)‡	1616 (811)	1117 (324)	0.005
	Gynoid (g)	4719 (1359)	3815 (638)	0.082
				
Fat Mass Ratio				
	Android/Gynoid	0.330 (0.107)	0.298 (0.098)	0.449
	Upper-body/Lower-body	1.346 (0.313)	1.229 (0.237)	0.330
	Central/Peripheral	0.922 (0.194)	0.711 (0.321)	0.004
Changes in other tendons (y:n)§		7:135	2:2	0.019

† - Variable not normally distributed, Mann-Whitney U test.

‡ - Normally distributed but significant difference in variance between groups.

§ - Expected cell count < 5, Fisher's Exact test.

In keeping with their lighter weight, the subjects with abnormal Achilles tendons had less total fat [all measured in grams] (17196 ± 3173, 21626 ± 7882, p = 0.009), trunk fat (7367 ± 1662, 10087 ± 4152, p = 0.003) and android fat (1117 ± 324, 1616 ± 811, p = 0.005). In contrast to the men, the women with Achilles tendon pathology had lower central/peripheral fat mass ratio (0.711 ± 0.321, 0.922 ± 0.194, p = 0.004) than women with normal tendons (Table 3).

Women with Achilles tendon pathology were more likely to have pathology affecting either the patellar or supraspinatus tendon than women with normal Achilles tendons (2/4 [50%], 7/142 [5%], p = 0.019) (Table 3).

**Figure 3:**
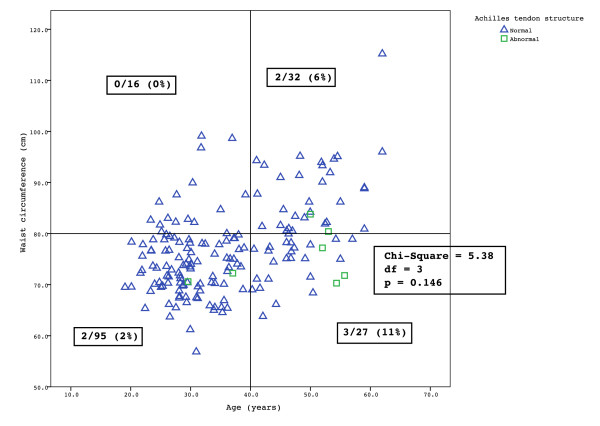
**Scatter-plot of age and waist circumference in women**. Women with normal tendons are indicated by blue triangles while women with asymptomatic Achilles tendon pathology are indicated by green squares.

The scatterplot of age versus waist did not show a clustering of women with abnormal tendons into any particular group (χ^2 ^= 5.375, df = 3, p = 0.1462) (Figure 3). The prevalence of tendon pathology among the four groups ranged from 0% to 11%.

## Discussion

Tendon pathology has been shown to be associated with fat distribution in two previous investigations; however, in both cases the study group included participants who were both symptomatic and asymptomatic [6,7]. As a consequence these findings may have been affected by altered physical activity behaviour secondary to tendon pain. Using anthropometry and DXA this investigation has shown that asymptomatic Achilles tendon pathology was associated with unique patterns of adipose tissue distribution but with quite different patterns in men and women. The changes in fat distribution were associated with tendon pathology in participants without a current or recent history of tendon pain. This removed the confounding effect of tendon pain changing physical activity behaviour and altering adiposity. As such, a strong case is made for body composition playing a role in the development of Achilles tendon pathology. This novel finding increases our currently limited knowledge surrounding the aetiology of Achilles tendinopathy.

Among the males in this cohort, despite no differences in mean height, weight, BMI, waist or hip circumference between the groups, the individuals with asymptomatic Achilles tendon pathology had significantly elevated WHR. This finding may be compared to that of Malliaras et al. [6] who found significant differences between WHR among three groupings of patellar tendon status -- normal tendons, unilateral imaging changes and bilateral imaging changes. The results of Malliaras et al. agree with our findings, in that, the group with normal tendons had the lowest WHR. The average WHR ratio among those with normal tendons was higher in our study (88.4 ± 9.8 cm Vs 79.1 ± 7.1 cm), most likely attributable to Malliaras' cohort being young (26.1 ± 5.3 years) athletes.

In contrast, while Malliaras et al. [6] found men with a waist circumference above 83 cm were much more likely to have patellar tendon abnormality, in this investigation waist circumference did not differ according to tendon status. However, individuals with asymptomatic Achilles tendon pathology were significantly older than those with normal tendons. This was the case for both men (15 years older) and women (11 years older), and is an aspect of the study that should be borne in mind when interpreting the body composition findings as adiposity generally increases with age [25]. We examined the interaction between age and waist circumference to explore these issues. Among the men there was a clustering of those with abnormal tendons into the group that was older than 40 years and with a waist circumference above 83 cm.

We found no difference in waist circumference or WHR between women with asymptomatic Achilles tendon pathology and those with normal tendons, again in agreement with data from Malliaras et al. [6]. This implies that some transference of body composition research is possible between the Achilles and the patellar tendon. Similarity in underlying mechanisms may account for the concordance of results [26]. Additionally, there was no significant clustering of women with abnormal tendons into any of the four groups constructed by the age and waist circumference cut-offs.

The men with abnormal Achilles tendons had higher android/gynoid fat mass ratios as well as higher upper-body/lower-body fat mass ratios. Expansion of adipose tissue depots in the upper body and, in particular the abdominal area, is associated with metabolic dysfunction and increased cardiovascular risk [11,27,28]. As both lipids and insulin resistance play a central role in this relationship, recent findings associating dyslipidaemia and Achilles tendinopathy [9] implicate abdominal fat in the pathoaetiology of Achilles tendinopathy among men. It is currently unclear whether this may occur though the consequences of increased abdominal adiposity (i.e. dyslipidaemia or insulin resistance) or some proximal factor that may contribute to abdominal fat storage (i.e. genetics, diet, behaviour, lipid processing).

Paradoxically, the opposite findings were seen in the women -- those with tendon abnormality had lower central/peripheral fat mass ratios. This finding is less likely to be related to blood lipid or insulin resistance as an underlying mechanism. A more likely explanation may be related to the effect that oestrogen has on body fat distribution. Among women, physiological levels of oestrogen prevent a central accumulation of adipose tissue. Oestradiol -- the main human oestrogen -- has been shown to inversely correlate with the central/peripheral fat ratio measured by DXA [29]. Both endogenous [30] and synthetic oestrogens [31,32] affect the function and metabolism of tendons. However, the long-term effects of menstruation, contraception and menopause on tendon are incompletely understood [33]. As oestrogen was not measured in this study we cannot speculate further, although we did note that the majority (63%) of the women with tendon pathology reported that they were either peri-menopausal or post-menopausal (Table 3).

Limitations of the current research must be acknowledged. First, data from two locations were pooled. Confounding was minimised by ensuring that data collection was identical wherever possible, and that approximately equal numbers of men and women as well as equal proportions of cases and controls were recruited from each location. Second, as all participants were asymptomatic it is unclear how these results relate to the clinical condition of Achilles tendinopathy. This is particularly pertinent given the complex relationship between tendon pathology and tendon pain [34]. The inclusion of asymptomatic participants was a deliberate action designed to eliminate the confounding effect that pain may have in modifying physical activity behaviour, which would lead to weight gain and invalidate the results. Third, although a large cohort was studied there were relatively few cases with tendon pathology. This limitation is a trade off related to the study design of only including individuals with no tendon pain.

Strengths of this investigation should also be highlighted. First, neither the participants nor the researchers knew tendon status when the individual volunteered for the study -- thus selection bias was limited. Second, during data collection personnel measuring anthropometry and performing DXA were not aware of tendon status. Third, this study was conducted in a large cohort of both men and women. Fourth, the accurate DXA technique was supplemented with inexpensive and readily available anthropometric measurements.

## Conclusion

In this large cohort, Achilles tendon pathology was associated with unique fat patterning in both men and women. Men with asymptomatic tendon pathology had a central distribution of fat, while women with asymptomatic tendon pathology had a peripheral distribution of fat in comparison to their peers with normal tendons. As all participants were free of tendon pain this is the best evidence to date that differences in adipose tissue distribution precede tendon pain.

### What is known

Achilles tendinopathy is a difficult to treat condition that presents not only in athletes but also in sedentary individuals. Cases in non-athletic individuals remain hard to explain through biomechanical theories. A central distribution of body fat has been linked with symptomatic patellar and lateral elbow tendinopathy. A limitation of these studies is that tendon pain may reduce physical activity and lead to weight gain, confounding the results. Studying asymptomatic populations and using ultrasound to define tendon status can avoid this limitation. Data addressing body fat distribution in relation to the Achilles tendon is lacking.

### What this study adds

Among a large cohort, this study showed that body fat distribution differed according to tendon status in asymptomatic participants. The men with tendon pathology showed a central distribution of body fat consistent with our hypothesis. By contrast, the women with tendon pathology had a peripheral distribution of body fat. An interaction between age and waist circumference was observed among men -- the highest prevalence of tendon pathology was among men older than 40 years and with a waist circumference >83 cm.

## Abbreviations

BMI: body mass index; DXA: dual-energy x-ray absorptiometry; ROI: region of interest; WHR: waist to hip ratio.

## Competing interests

The authors declare that they have no competing interests.

## Authors' contributions

JEG participated in the study design, acquisition of data, analysis and interpretation of data, manuscript preparation and statistical analysis. HA participated in the study design, acquisition of data, analysis and interpretation of the data and manuscript preparation. ZSK participated in the study design, acquisition of data and manuscript preparation. SLB participated in the study design, analysis and interpretation of data and manuscript preparation. JCL participated in the study design, acquisition of data, analysis and interpretation of data, manuscript preparation and statistical analysis. All authors have read and approve the final version of the manuscript.

## Pre-publication history

The pre-publication history for this paper can be accessed here:

http://www.biomedcentral.com/1471-2474/11/41/prepub
